# Domination and power domination in a one-pentagonal carbon nanocone structure

**DOI:** 10.3389/fchem.2024.1423055

**Published:** 2024-06-20

**Authors:** Shoba Pandian, Mohana N.

**Affiliations:** Department of Mathematics, School of Advanced Sciences, Vellore Institute of Technology, Chennai, India

**Keywords:** chemical graph, one-pentagonal carbon nanocone, domination, power domination, brick diagram

## Abstract

Domination is an important factor in determining the robustness of a graph structure. A thorough examination of the graph’s topological structure is necessary for analyzing and examining it for various aspects. Understanding the stability of a chemical compound is a significant criterion in chemistry, which necessitates conducting numerous experimental tests. The domination number and power domination number are pivotal in defining a wide range of physical properties, which include physiochemical properties, thermodynamic properties, chemical activities, and biological activities. The one-pentagonal carbon nanocone (1-PCNC) is a member of the carbon nanocone family and has a structure similar to that of honeycomb networks, which are renowned for their robustness. In this paper, we find the domination number and power domination number of 1-PCNC by considering it as an (*m*-1)-layered infinite graph.

## 1 Introduction

In the field of nanotechnology, the study of carbon-based nanomaterials like fullerenes, carbon nanotubes, carbon nanohorns, carbon nanowires, and carbon nanocones using a graph theoretical approach is becoming very intense and popular because of its application in the real world. Carbon nanocones (CNCs) are gaining priority in the field of research because of their applications in multidisciplinary areas. The study and practical application of domination and its variants have attracted significant attention, particularly in the realm of neural networks (Prabhu et al., 2022) and in the family of chemical graphs (Vukičević and Klobučar, 2007; Quadras et al., 2015; Gao et al., 2018; Hutchinson et al., 2018; Chithra and Menon, 2020; Iqbal et al., 2020; Prabhu et al., 2021). Researchers have discovered that chemical graphs offer significant insights into molecular structure, reactivity, and other characteristics, which allow for the prediction of molecular behavior and facilitate the creation of new compounds with specific properties. The application of the domination number in the analysis of secondary RNA structure (Haynes et al., 2006) and the encryption of bit strings into a DNA sequence (Yamuna and Karthika, 2014) has given rise to numerous research avenues within the domain of chemical graph theory. The chemical graph theory involves analyzing chemical structures by representing them as graphs, in which vertices correspond to the atoms of the chemical compound, while the edges represent the bonds between the atoms. QSAR and QSPR investigations have consistently revealed the significant association between graph theoretical invariants, commonly referred to as topological indices or molecular descriptors, and a wide range of physical and chemical properties exhibited by molecules. To examine the physiochemical characteristics of a molecule, it is crucial to conduct a comprehensive analysis of its topological indices or molecular descriptors (Zaman et al., 2023; Ullah et al., 2024; Zaman et al., 2024). The Wiener index, classified as a topological index, for 1-PCNC was determined by employing JAVA programming (Alipour and Ashrafi, 2009). Recently, the topological properties of carbon nanocones, employing the cut technique on strength-weighted graphs to identify various topological indices, were investigated and studied. Using this methodology, analytical formulations for numerous distance-based topological indices of CNCs were derived (Arockiaraj et al., 2018). To find the desired sizes and chemical reactivity for 1-PCNC, topological modeling methods have been used (Bultheel and Ori, 2018). In contrast to the Weiner index, the edge-Wiener index plays a crucial role in molecular analysis. The edge-Wiener and vertex-edge-Wiener indices were specifically investigated in coronoid systems, carbon nanocones, and SiO_2_ nanostructures. This examination included breaking down the original graph into smaller strength-weighted quotient graphs using the Djoković–Winkler relation (Arockiaraj et al., 2019b). A computational method was employed to calculate the Mostar, edge-Mostar, and total-Mostar indices by considering the strength-weighted parameters. These techniques were then applied to determine the three indices for various coronoid and carbon nanocone structures (Arockiaraj et al., 2019a). Expanding the study further on 1-PCNC, the edge metric dimension and the bounds on the partition dimension of the 1-PCNC structure were calculated (Sharma et al., 2021; Koam et al., 2022). Carbon-based nanomaterials are characterized by their remarkable flexibility and strength, making them well-suited for manipulating various nanoscale structures. This unique property suggests that carbon nanomaterials will be instrumental in advancing the field of nanotechnology engineering. The development of 3D all-carbon architectures has the potential to revolutionize power storage, field emission transistors, photovoltaic systems, supercapacitors, biomedical implants, and high-performance catalysts (Sharma et al., 2021). We delve further into this research endeavor because of its immense practicality and widespread implementation in various areas. We explore the domination and power domination of 1-PCNC by examining its brick diagram, akin to that of n-layered honeycomb networks (HC(n)) (Stojmenovic, 1997). This paper provides an overview of the intricate layout of the brick diagram of 1-PCNC, along with the precise calculations of the domination number, independent domination number, power domination number, and *k*-power domination number.

## 2 Preliminaries

A graph *G* is represented as an ordered pair [V(*G*), E(*G*)], where V(*G*) denotes a non-empty set of vertices and E(*G*) represents a set of unordered pairs of distinct elements from V(*G*), which are known as edges. A subgraph *S* of a graph *G* is essentially a graph where the set of vertices V(*S*) is a subset of V(*G*) and the set of edges E(*S*) is a subset of E(*G*). The order of the graph *G* is characterized by the total count of vertices within *G*. Simultaneously, the size of the graph *G* is determined by the total count of edges present in *G*. The count of edges connected to a vertex *x* is termed the degree of the vertex, represented by deg(*x*). The maximum degree within the graph is symbolized as Δ. The distance between two vertices within a graph refers to the count of edges present in the shortest path connecting them. The collection of vertices adjacent to *x* is known as the neighborhood of *x*, symbolized as *N*(*x*), and *N*[*x*] = *N*(*x*) ∪{*x*} is called the closed neighborhood of vertex *x*. A cut vertex is a vertex *x* ∈ *V* such that G \{*x*} disconnects the graph *G*. Assigning a dominating vertex to each vertex in a graph such that every vertex is dominated exactly once is called saturation. A vertex with degree 1 is commonly referred to as a pendant vertex, and if one of the vertices of an edge is a pendant vertex, then the edge is called a pendant edge. A cut *C* denoted as (*T*, *H*) is a partition of the vertex set *V* in a graph *G* into two subsets, *T* and *H*. The cut-set of a cut *C* = (*T*, *H*) is represented by the set {(*x*, *y*) ∈ *E*|*x* ∈ *T*, *y* ∈ *H*}, which includes edges having one endpoint in the set *T* and the other endpoint in the set *H*. A hexagonal system is a finitely connected planar network with no cut vertices, where every inner area is a regular hexagon that is mutually congruent. A hexagonal chain (HC) is described as a hexagonal arrangement in which each hexagon is adjacent to a maximum of two other hexagons, and if every shared edge between two adjacent hexagons is parallel to the other, the HC is said to be linear. A hexagon in an HC is a linear hexagon *h* if it has two inner vertices in different lines. A unique vertex *y* that is situated at a distance of 3 from the specified vertex *x* in a hexagon is called the diagonally opposite vertex of *x*.

A dominating set for a graph *G* is a subset *D* of vertices where each vertex not in *D* is adjacent to at least one vertex in *D*. The domination number *γ*(*G*) signifies the count of vertices in the smallest dominating set for *G*. A dominating set is said to be an independent dominating set when no two vertices within the set are adjacent, and the independent domination number, *γ*_*i*_(*G*), expresses the minimum cardinality of such a set. A dominating set *D* is said to be a power dominating set if every vertex in *G* is dominated by *D* concerning the following domination rule: (a) every vertex incident on a dominated edge is dominated. (b) Every edge joining two dominated vertices is dominated. (c) If a vertex is connected to *i* > 1 edges and *i* − 1 of these edges are dominated, then all *i* of these edges must be dominated, and the power domination number, denoted by *γ*_*p*_(*G*), indicates the smallest cardinality of such a set. A dominating set *D* is said to be a *k*-power dominating set if every vertex in *G* is dominated by *D* concerning the following domination rule: (a) every vertex incident to a dominated edge is dominated. (b) Every edge joining two dominated vertices is dominated. (c) If a vertex is connected to *i* > *k* edges and *i* − *k* of these edges are dominated, then all *i* of these edges must be dominated, and the *k*-power domination number, *γ*_*p*,*k*_(*G*), indicates the smallest cardinality of such a set.

Theorem 1 (Berge, 1973) In a graph G with order *m*, *m ≥ 1* and maximum degree Δ, it is established that the domination number *γ(G)* satisfies $\gamma \left(G\right)\geq \frac{m}{\Delta +1}$ .

Proposition 1 (Bermudo et al., 2020) If H is a hexagonal chain and D is a minimum dominating set in H, then every linear hexagon in H contains at least two vertices of D.

Theorem 2 (Bermudo et al., 2020) Suppose *H* is a hexagonal chain comprising *h* hexagons where *h* ≥1 , then the domination number γ(*H*) satisfies the inequality $h+1\leq \gamma \left(H\right)\leq h+\left\lceil \frac{h}{3}\right\rceil$ .

Proposition 2 (Bermudo et al., 2020) Suppose H is a hexagonal chain comprising h hexagons where h ≥1 , then γ(H) = h + 1.

Theorem 3 (Alanko et al., 2011) For a grid graph G = (V, E), with order *m* × *n*, suppose *S*_1_ ⊆ *V* and *S*_2_ ⊆ *V* with |*S*_1_| = |*S*_2_| and *D*(*S*_1_) ⊆ *D*(*S*_2_). To determine a minimum dominating set of G, it is sufficient to exclude *S*_1_ and focus solely on dominating sets that extend *S*_2_.

Lemma 1 (Chang et al., 2012) *Consider a connected graph G with a maximum degree*, Δ(*G*) ≤ *k* + 1, *for* *k* ≥ 1 *then* *γ*_*p*,*k*_ = 1.

## 3 Address of blocks in the brick diagram

**FIGURE 1:**
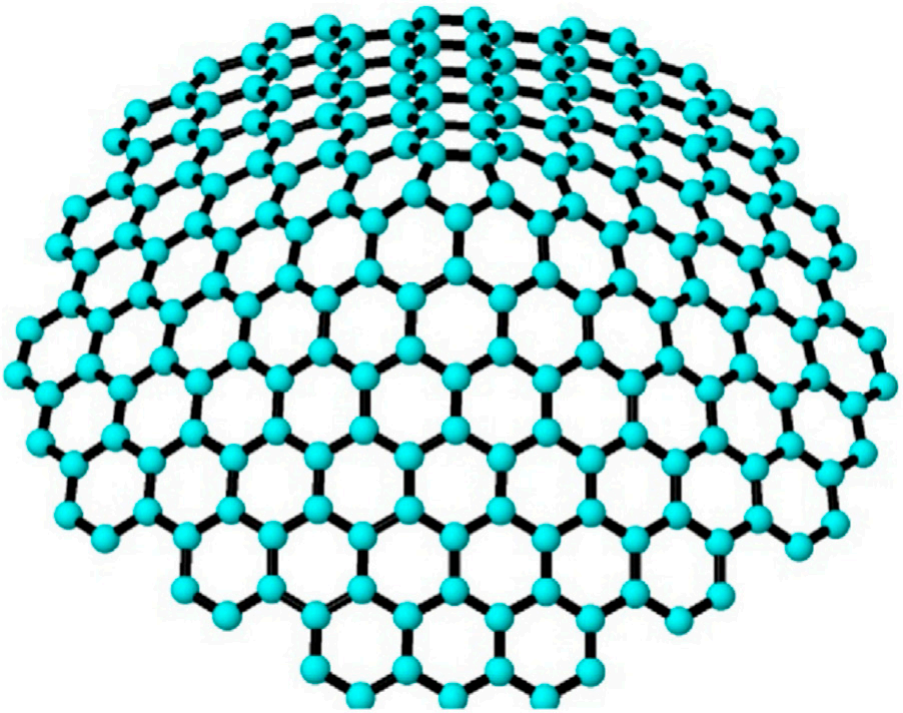
Pentagonal nanocone structure (Sharma et al., 2021).

CNCs consist of an interconnected arrangement of carbon atoms in a hexagonal lattice structure. CNCs were first synthesized in 1994 (Ge and Sattler, 1994), in which the distinct hollow carbon structures, identified as carbon cones, were observed on a flat graphite surface alongside tubules. The chemical graph of CNCs denoted by *CNC*_*t*_[*m*] with *m* ≥ 2 exhibits a tapered structure with edges and vertices arranged in a specific pattern, and at its core, there is a cycle of size with the same order *t*. Surrounding this central cycle, there are (*m*-1) hexagon layers forming the tapered surface. CNCs are carbon frameworks that can be depicted as infinite cubic plane graphs featuring 1 ≤ P ≤5 pentagons alongside hexagons forming the rest of the faces (Brinkmann and Van Cleemput, 2011). The 5-P seed (five pentagons at the apex) exhibits a shape that closely resembles that of a sphere, making it the most similar to a spherical shape among the five available seed options. Consequently, the growth of a graphitic network can proceed smoothly from the 5-P seed, as there is minimal strain in the transition region. On the other hand, the 2-P, 3-P, and 4-P seeds possess non-spherical shapes, which would result in higher strain when attempting to match their corresponding cones. As a result, the formation of these cones is unlikely. The 1-P seed showcases an intriguing characteristic of effortlessly producing a cone, without experiencing any strain (Ge and Sattler, 1994). Its resemblance to honeycomb networks serves as a compelling motivation for further exploration into its topological robustness. In this paper, we study the case for *t* = 5, specifically the *CNC*_5_[*m*] with *m* ≥ 2, i.e., the 1-PCNC structure which belongs to the family of CNCs and gained its identity in the year 1994 (Ge and Sattler, 1994). The 1-PCNC is produced by removing a 60° wedge from a graphene structure and connecting the edges to create a nanocone featuring a singular pentagonal defect at its apex. Inserting a pentagon into the honeycomb layer introduces a disclination defect in the graphenic plane, leading to the formation of a tapered-like structure with a positive curvature. This structure encloses one pentagon within the first belt of five hexagons, where the pentagon is called the network basis (core). The chemical structure of a 1-PCNC is depicted in Figure 1. Figures 3A, B show a pentagon and *CNC*_5_[2], which is obtained by expanding five hexagons to the outer borders of a pentagon. *CNC*_5_[*m*] is generated recursively by inserting a layer of hexagons to the outside edges of *CNC*_5_[*m* − 1].

**FIGURE 2:**
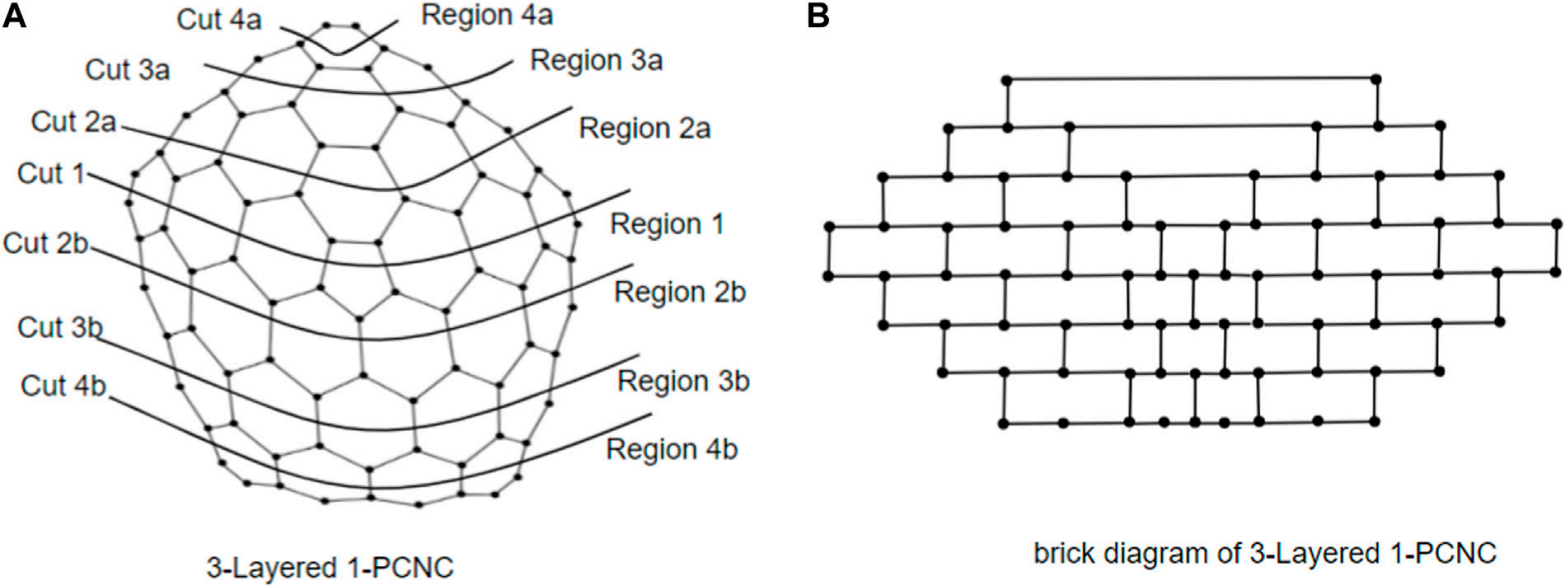
**(A)** 3-Layered *CNC*_5_[4]; **(B)** brick diagram of 3-Layered 1-PCNC.

Now, we model a brick diagram of *CNC*_5_[*m*], *m* ≥ 2, which facilitates our understanding of the 1-PCNC structure. Initially, our approach involves depicting a series of cuts on the graph, starting from Region 1 and extending downward until all regions are encompassed. These cuts are systematically labeled to denote their origin and direction. For instance, in Figure 2A, we identify the cut surrounding the core of *CNC*_5_ [4] within Region 1 as “cut 1.” Moving onward, as we ascend into Region 2a, we establish a new cut labeled “cut 2a.” This pattern continues as we progress through the regions, with each successive cut being denoted by appending the region identifier to the numeric label (e.g., “cut 4a” in Region 4a). As we descend from Region 1, we continue the process of defining cuts, assigning labels such as “cut 2b,” “cut 3b,” and “cut 4b” in a manner consistent with our established pattern. Each cut serves to delineate a boundary within the graph, segregating regions and defining the interconnected structure. In the brick diagram representation, the effect of these cuts becomes visually apparent. A straight horizontal line emerges when extending the zigzag lines above and below each cut. The edges intersecting with these cut lines correspond to the vertical lines depicted in the brick diagram, while the regions depicted by the cuts form distinct blocks within the diagram (as illustrated in Figure 2B).

**FIGURE 3:**
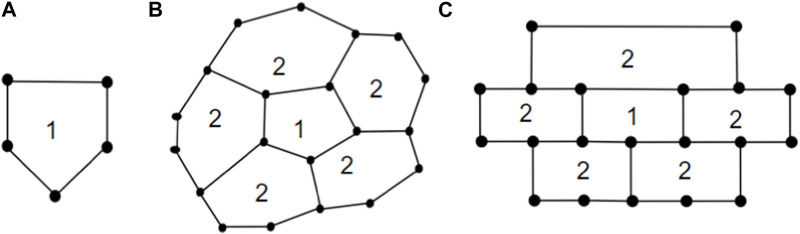
**(A)** One Pentagon; **(B)** 1-layered *CNC*_5_[2]; **(C)** brick diagram of 1-layered *CNC*_5_[2].

**FIGURE 4:**
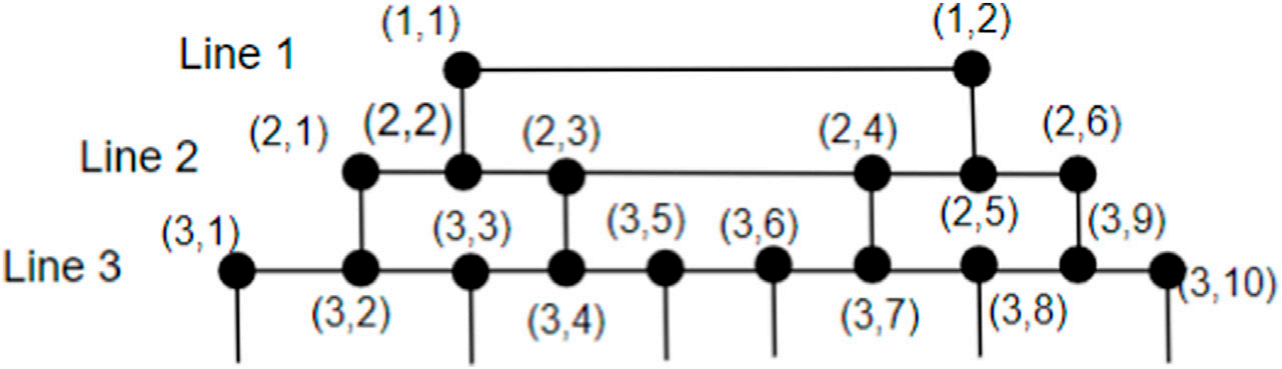
Positioning nodes in the brick diagram.

**FIGURE 5:**
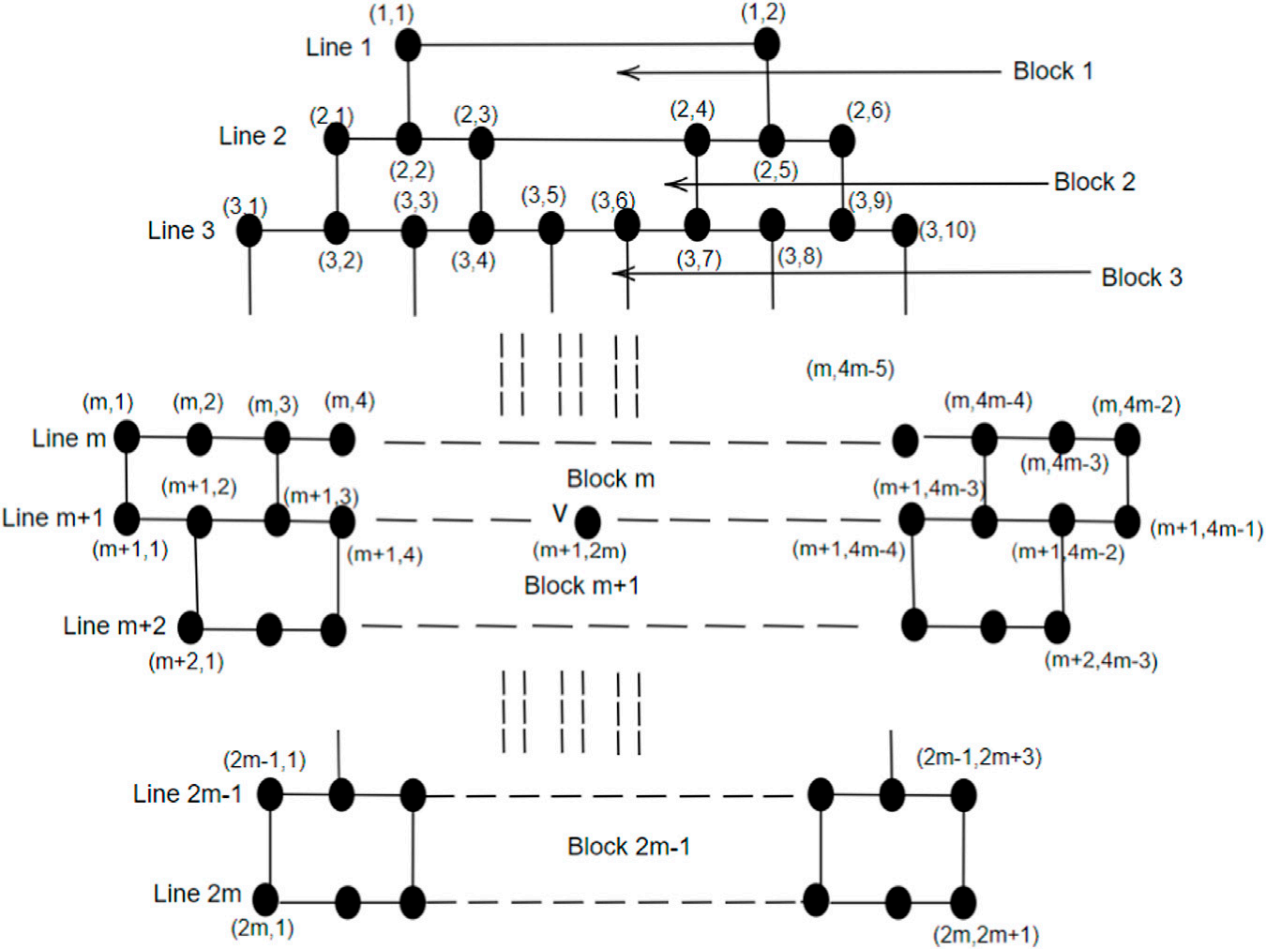
Addressing of Blocks in the brick diagram of *CNC*_5_[*m*].

To simplify the visualization of a *CNC*_5_[2], we may identify it using its brick diagram, as shown in Figure 3C. A higher-level 1-PCNC network is generated by expanding a lower-level network using a level numbering approach similar to the method that was used to build honeycomb networks (Sharieh et al., 2008). In the context of *CNC*_5_[*m*], where *m* ≥ 2, the quantity 5*m* ^2^ denotes the number of vertices, and $5\left(m^{2}+\frac{m\left(m-1\right)}{2}\right)$ represents the number of edges. Additionally, there are $\frac{5m\left(m-1\right)}{2}$ hexagons alongside one pentagon within this structure. Each node in *CNC*_5_[*m*] can be expressed in the form of (*i*, *j*), where *i* denotes the node’s presence in line *i* and *j* denotes the node’s location in line *i*, as depicted in Figure 4. The vertex (1, 1) serves as an example of how to identify the first node in line 1. The vertices on line *m* are represented by (*m*, 1), (*m*, 2), (*m*, 3),…, (*m*, 4*m*-5), (*m*, 4*m*-4), (*m*, 4*m*-3), and (*m*, 4*m*-2). The vertices on lines *m* + 1 and *m* + 2 may be represented by (*m* + 1, 1), (*m* + 1, 2), (*m* + 1, 3),…(*m* + 1, 2*m*),…(*m* + 1, 4*m* − 4), (*m* + 1, 4*m* − 3), (*m* + 1, 4*m* − 2), (*m* + 1, 4*m* − 1) and (*m* + 2, 1), (*m* + 2, 2), (*m* + 2, 3),…(*m* + 2, 4*m* − 5), (*m* + 2, 4*m* − 4), (*m* + 2, 4*m* − 3). The graph *CNC*_5_[*m*] consists of 2*m* lines, and the vertices in line 2*m* are represented by (2*m*, 1), (2*m*, 2), (2*m*, 3),…, (2*m*, 2*m*), (2*m*, 2*m* + 1), depicted in Figure 5. The Block *i*, 1 ≤ *i* ≤ *m* − 1, denotes the number of hexagons in each Block and identifies the subgraph induced by line *i* and line *i*+1 and is defined by Block $i=\left\{\begin{array}{cc} 2i-1, & i=1,2,3,\ldots ,m-1\\ 2i-2, & i=m,\\ 2m-n-1, & 1\leq n\leq m-1,m+n\leq i\leq 2m-1 \end{array}\right.$

## 4 Main result

In this section, we compute the domination number, independent domination number, power domination number, and *k*-power domination number for 1-PCNC. To streamline the explanation of the lemmas, we introduce certain notations that prove to be beneficial for the subsequent discussion.

• For any *x* ∈ *D*, *A*(*x*) denotes the collection of vertices adjacent to *x*.
• For a set *S* ⊆ *V*, the collection of vertices adjacent to *S* is denoted by *A*(*S*).
• In a brick diagram of *CNC*_5_[*m*], the Block *m* is called a 1-pentagonal hexagonal chain and denoted by 1-PH.
• In a linear hexagonal chain, if there are exactly two pendant edges attached to the first and last hexagon, we define it as a 2-pendant linear hexagonal chain, denoted by 2P-HC.

Observation. Let G = *CNC*_5_[*m*] be a connected undirected graph, *m* ≥ 2. Then, we have the following observations.

1. In the *CNC*_5_[*m*] graph, there are 5*m* vertices with degree 2, while the remaining vertices have a degree 3.
2. The last Block, *B*_2*m*−1_, consists of exactly *m* number of hexagons.
3. From line *L*_1_ to line *L*_*m*_, the number of vertices is increased by 4; from line *L*_*m*_ to line *L*_*m*+1_, the number of vertices is increased by 1; and from line *L*_*m*+1_ to line *L*_2*m*_, the number of vertices is decreased by 2.
4. The diameter of *CNC*_5_[*m*] is 4*m*-2.

Proof: The first, second, and third observations can be easily deduced from the structure of 1-PCNC. However, to establish the validity of the fourth observation, we employ mathematical induction. Within the core of 1-PCNC, there exists a pentagon where the distance between any two nodes within the pentagon is less than or equal to 2. Now, let us assume that the distance between any two nodes in *CNC*_5_[*m* − 1] is less than or equal to 4(*m* − 1) − 2. Each node situated on the boundary of *CNC*_5_[*m*] is at a distance of either 1 or 2 from a node belonging to *CNC*_5_[*m* − 1]. Consider two nodes, *u* and *v*, in *CNC*_5_[*m*], and let |*uv*| represent the distance between nodes *u* and *v*. We can find two nodes, *u*′ and *v*′, from *CNC*_5_[*m* − 1] such that |*uu*′| ≤ 2 and |*vv*′| ≤ 2. Consequently, we have |*uv*| ≤ |*uu*′| + |*u*′*v*′| + |*v*′*v*| ≤ 4*m* − 2.

Lemma 2 In G = *CNC*_5_[*m*], every vertex *v* ∈ *V*(*G*) is dominated exactly once when *m* is even.

**FIGURE 6:**
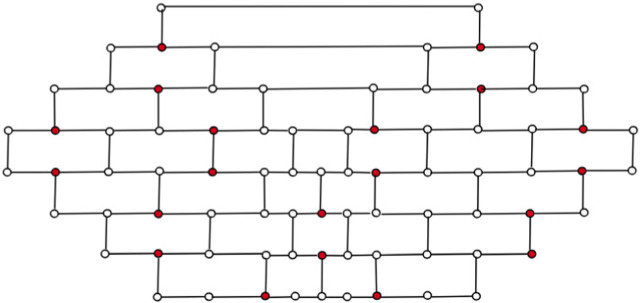
*γ*(*CNC*_5_[4]) = 20.

Proof. By constructing an *m*-layered *CNC*_5_[*m*] graph recursively, one may build hexagonal layers in a circular pattern around a one pentagon. The number of vertices of degree 2 is 5*m*, and the count of vertices with a degree of 3 is 5*m*(*m* − 1). When *m* is even, one can symmetrically select exactly $\frac{5m^{2}}{4}$ vertices of order 3 such that all the graph’s vertices are dominated exactly once. This is evident from the inherent structure of graph *G*. For instance, in Figure 6, the vertices highlighted in red are the dominating set of the graph, *CNC*_5_ [4].

Lemma 3 Let G=2P-HC. Then, $\gamma \left(G\right)=\left\{\begin{array}{cc} h+2, & h\;\text{is odd number of hexagons},\\ h+1, & h\;\text{is even number of hexagons} \end{array}\right.$

**FIGURE 7:**
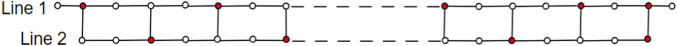
Odd number of hexagons.

Proof. **When** *h* **is odd:** from proposition 2, we know that the domination number for a linear HC is *h*+1. Now, in 2P-HC, we begin by choosing the second vertex of line 1 and proceed by selecting the diagonally opposite vertices such that all the vertices of the hexagons are dominated (with exactly two vertices) except the pendant vertex of the pendant edge attached to the last hexagon. We must choose the second-last vertex from line 1 to dominate the remaining pendent vertex. Hence, the number of vertices selected to dominate the 2P-HC is *h*+2, which is depicted in Figure 7.

**FIGURE 8:**
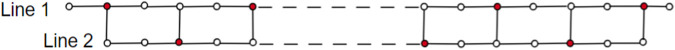
Even number of hexagons.

**When** *h* **is even:** we begin by choosing the second vertex of line 1 in 2P-HC and proceed by selecting the diagonally opposite vertices from it, such that all the vertices of the hexagons are dominated. Hence, the number of vertices selected to dominate the 2P-HC is *h*+1, as represented in Figure 8.

Lemma 4 Consider *H* as a subgraph of G = CNC_5_[*m*], which consists of lines up to (*m* − 1), where *m* is odd. Then, $\gamma \left(H\right)=2{\left(\frac{m-1}{2}\right)}^{2}$ .

**FIGURE 9:**
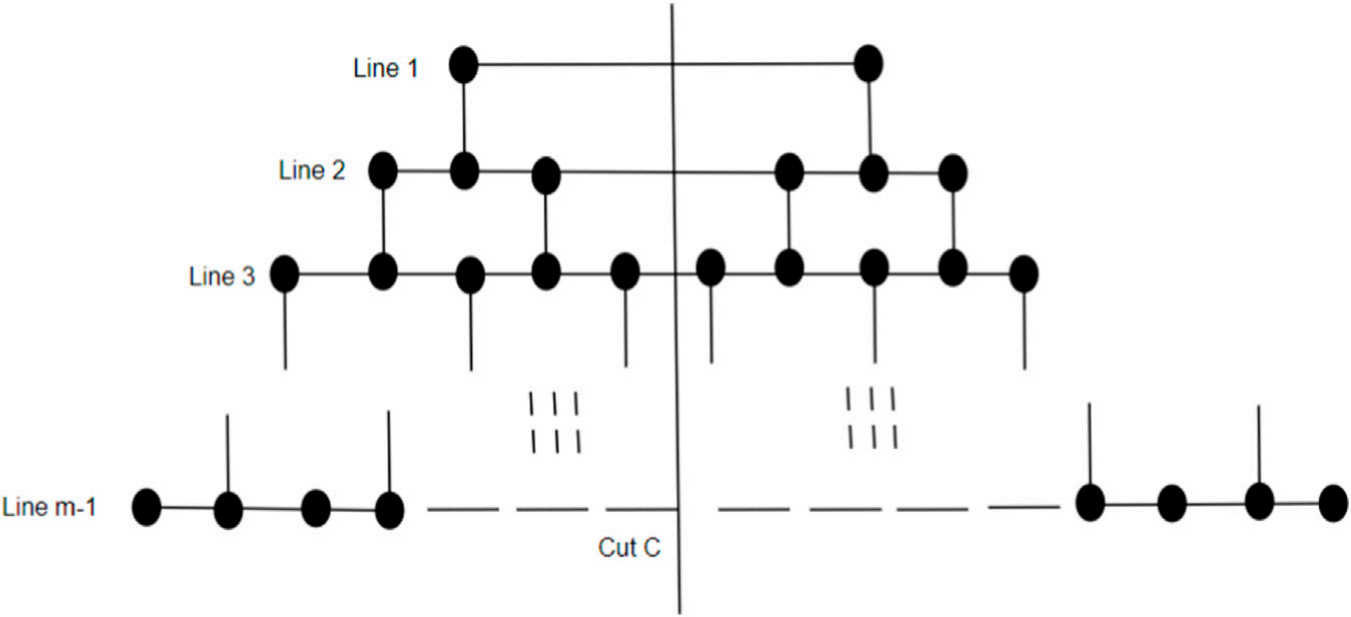
Subgraph H.

Proof. We subdivide the subgraph *H* as shown in Figure 9 into two partitions by making a cut C = (*S, T*), where *S* contains 2*i* − 1 vertices from each line *i*, 1 ≤ *i* ≤ (*m* − 1). Two vertices *v*_*i*_ and *v*_*j*_ in *S* are connected if and only if the edge *v*_*i*_ *v*_*j*_ ∈ *E*(*H*). It is enough to dominate *S*, as *T* is the mirror image of *S* and follows an equal number of vertices to constitute the same dominating set as that of *S*. To dominate *S*, we select vertices line-wise, dominating one vertex of line 1 by choosing exactly one vertex with address (2, 2). Proceeding by selecting the diagonally opposite vertices up to line (*m* − 1) such that all the vertices are dominated exactly once, in general, we get the following: $\gamma \left(S\right)=\gamma \left(T\right)={\left(\frac{m-1}{2}\right)}^{2}$ as shown in Table 1 which depicts the domination number of a partition *H* upto line (*m*-1) where *m* is greater than or equal to 3. Hence, the domination number of *H* is given by $\gamma \left(H\right)=\gamma \left(S\right)+\gamma \left(T\right)=2{\left(\frac{m-1}{2}\right)}^{2}$ .

**TABLE 1:** Domination of a partition *H*.

*m*	3	5	7	.	.	.	*n*
*γ*(*S*)	1^2^	2^2^	3^2^	.		.	${\left(\frac{n-1}{2}\right)}^{2}$

Lemma 5 Consider G = *CNC*_5_[*m*] as a connected undirected graph *m* ≥ 2. Then, we have *γ*(1-PH) = 2*m*.

Proof. In 1-PH, the number of hexagons is 2*m* − 2, arranged with the pentagon at the center, flanked by (*m* − 1) hexagons on each side. By proposition 2 to dominate the hexagons on the left side, we require *m* vertices. Similarly, to dominate the hexagons on the right side, another set of *m* vertices is needed. Therefore, a total of 2*m* vertices are required to dominate the entire 1-PH.

Theorem 4 Consider G = *CNC*_5_[*m*] as a connected undirected graph m ≥ 2. Then, we have

$\gamma \left(G\right)=\left\{\begin{array}{cc} \frac{5m^{2}}{4}, & m\;\text{is even},\\ \left\lceil \frac{5m^{2}}{4}\right\rceil +1, & m\;\text{is odd} \end{array}\right.$

Proof. Case (i) When ***m is even***. By Lemma 2 and Theorem 1, it is straightforward that $\gamma \left(G\right)\geq \frac{p}{\Delta +1}=\frac{5m^{2}}{4}$ , where *p* is the order of the graph. Each vertex is dominated precisely once, starting from the initial vertex with address (2,2) and proceeding by saturating a sequence of diagonally opposite vertices of a hexagon till all the vertices are dominated.

Case (ii) When ***m is odd***. The *m*-layered *CNC*_5_[*m*] graph has 2*m* lines and 2*m* − 1 blocks. We consider three subgraphs: *H*_1_, *H*_2_, and *H*_3_. The subgraph *H*_1_ contains Block 1, Block 2, … Block (*m* − 2). The subgraph *H*_2_ contains Block *m*; the subgraph *H*_3_ contains Block (*m* + 2), Block (*m* + 3),…Block (2*m* − 1). Let *D* ⊆ *V* be a dominating set, and we select vertices from *H*_1_, *H*_2_, and *H*_3_ in a set *D* as follows:*H*_1_ consists of (*m*-1) lines and (*m*-2) blocks. Using Lemma 4, $\gamma \left(H_{1}\right)=2{\left(\frac{\left(m-1\right)}{2}\right)}^{2}$ .

The subgraph *H*_2_ consists of 1-PH, i.e., Block *m*, using Lemma 5, *γ*(1-PH) = 2*m*. Now, we consider the subgraph *H*_3_, and to find the dominating vertices of *H*_3_, we employ the depth-first search (DFS) algorithm, known for its exhaustive search approach, where |*H*_3_| is the input parameter, and it consists of lines *m* + 2 to 2*m*. To select vertices from |*H*_3_| in *D*, we first fix the root vertex as (*m* + 2, 2), and we maintain two sets *D*_1_ and *A* (*D*_1_), where *D*_1_ is the dominating set of *H*_3_. The algorithm gets terminated once |*A* (*D*_1_)| = |*H*_3_ − *D*_1_|. We begin by dominating the vertices of *H*_3_ in a sequential order of the index *i*, i.e., we proceed by covering all the vertices (*m* + *i*, 1) where 1 ≤ *i* ≤ *m* and continuing up to all the vertices (*m* + *i*, *j*), where 1 ≤ *j* ≤ 4*m* − (2*i* − 1) are dominated. We use Theorem 2, Theorem 3, Proposition 1, and Lemma 3 to select vertices in set *D*_1_ such that |*A* (*D*_1_)| = |*H*_3_ − *D*_1_|. $\gamma \left(H_{3}\right)=\frac{\left|H_{3}\right|}{4}+1$ .

Hence, the total number of vertices in D is $\frac{5m^{2}}{4}+1$ , which implies $\gamma \left(G\right)\leq \frac{5m^{2}}{4}+1$ .

**FIGURE 10:**
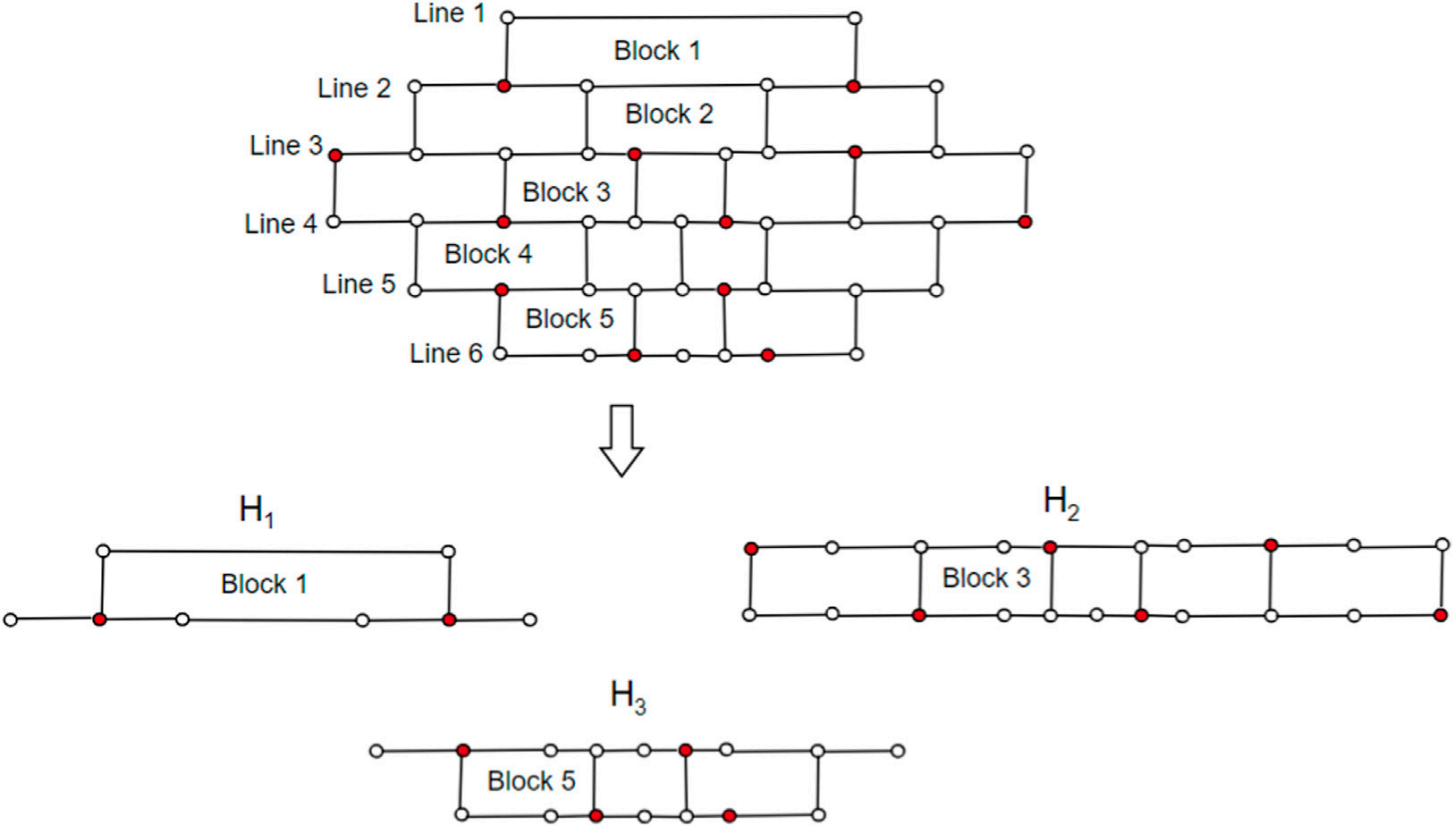
The brick diagram representation of *CNC*_5_[3].

On the other hand, to prove $\gamma \left(G\right)\geq \frac{5m^{2}}{4}+1$ , we use the contradiction method. Let us assume that $\gamma \left(G\right)=\left\lceil \frac{5m^{2}}{4}\right\rceil$ . Consider the graph *CNC*_5_ [3] as shown in Figure 10. We divide the graph into *H*_1_, *H*_2_, and *H*_3_. To dominate *H*_1_ in total, we need 2 ${\left(\frac{m-1}{2}\right)}^{2}=2$ vertices. To dominate all the vertices of the hexagons in *H*_2_ by Lemma 5, we need a minimum of 2*m* vertices, i.e., six vertices. To dominate *H*_3_, we need *h* + 2 = 5 vertices, by Lemma 3. Hence, *γ*(*G*) = 13, but according to our assumption, $\gamma \left(G\right)=\left\lceil \frac{5m^{2}}{4}\right\rceil =12$ , which is a contradiction to our assumption. Hence, $\gamma \left(G\right)\geq \left\lceil \frac{5m^{2}}{4}\right\rceil +1$ .

Theorem 4 establishes that the dominating set created by choosing vertices coincides with an independent set, which leads to the assertion of Theorem 5.

Theorem 5 Let G = CNC_5_ [m] as a connected undirected graph m ≥ 2. Then, we have

$\gamma_{i}\left(G\right)=\gamma \left(G\right)=\left\{\begin{array}{cc} \frac{5m^{2}}{4}, & m\;\text{is even},\\ \left\lceil \frac{5m^{2}}{4}\right\rceil +1, & m\;\text{is odd} \end{array}\right.$

Theorem 6 Let G = CNC_5_[m], m ≥ 2. Then, $\gamma_{p}\left(G\right)=\left\lceil \frac{2m}{3}\right\rceil$ .

Proof. Consider *D* to be a minimum power dominating set and *D*_1_ to be a power dominating set. When *m* = 2, it is easy to verify from the brick diagram that *D* = {(2, 2), (3, 3)} is a minimum power dominating set. Now, we prove for *m* ≥ 3. To prove $|D|\leq \left\lceil \frac{2m}{3}\right\rceil$ , the following cases are considered:

Case (i) when *m* = 3*i* for all *i* ≥ 1, consider *D*_1_ = (3*n* − 1, 4*n* − 2) ∪ (3*n*, 4*n* + 1), 1 ≤ *n* ≤ *i;* this implies that $|D_{1}|=2i=\frac{2m}{3}$ .
Case (ii) when *m* = 3*i* + 1 for all *i* ≥ 1, consider *D*_1_ = (3*n* − 1, 4*n* − 2) ∪ (3*n*, 4*n* + 1) ∪ (3*i* + 2, 4*i* + 1), 1 ≤ *n* ≤ *i;* this implies that $|D_{1}|=2i+1=\left\lceil \frac{2m}{3}\right\rceil$ .
Case (iii) when *m* = 3*i* + 2 for all *i* ≥ 1, consider *D*_1_ = (3*n* − 1, 4*n* − 2) ∪ (3*n*, 4*n* + 1) ∪ (3*i* + 2, 4*i* + 2) ∪ (3*i* + 3, 4*i* + 4), 1 ≤ *n* ≤ *i;* this implies that $|D_{1}|=2i+2=\left\lceil \frac{2m}{3}\right\rceil$ . Hence, $|D|\leq \left\lceil \frac{2m}{3}\right\rceil$ .

To prove $|D|\geq \left\lceil \frac{2m}{3}\right\rceil$ , we use the contradiction method. Let us assume that $|D|=\left\lceil \frac{2m}{3}\right\rceil -1$ . Now, we know that when *m* = 2, then |*D*| = 2, but according to our assumption |*D*| = 1. Hence, it is a contradiction. Thus, $|D|\geq \left\lceil \frac{2m}{3}\right\rceil$ which implies $\gamma_{p}\left(G\right)=\left\lceil \frac{2m}{3}\right\rceil$ .

Theorem 7 *Let G =* *CNC*_5_ [*m*], *m* ≥ 2. *Then*, *γ*_*p*,*k*_(*G*) = 1.

Proof. Since Δ(*G*) = 3 by Lemma 1, its straightforward.

## 5 Conclusion

In this research, we have conducted a thorough investigation of the 1-PCNC graph by analyzing different domination parameters. Modeling the brick diagram associated with 1-PCNC allowed us to identify several crucial parameters, including the domination number, independent domination number, power domination number, and *k*-power domination number. We believe that these findings will assist researchers in gaining insights into and predicting the physiochemical properties associated with these chemical structures. Moving forward, we aim to expand our work by understanding in depth the physiochemical properties of chemical structures using different variants of domination.

## Data Availability

The original contributions presented in the study are included in the article/supplementary material; further inquiries can be directed to the corresponding author.
